# PR3 vasculitis presenting with symptomatic splenic and renal infarction: a case report and literature review

**DOI:** 10.1186/s12882-019-1266-5

**Published:** 2019-03-06

**Authors:** M. J. Bottomley, M. Gibson, B. Alchi

**Affiliations:** 1 Department of Renal Medicine, Royal Berkshire Hospital, Royal Berkshire NHS Foundation Trust, Reading, UK; 2 Department of Radiology, Royal Berkshire Hospital, Royal Berkshire NHS Foundation Trust, Reading, UK

**Keywords:** Vasculitis, ANCA, PR3, Wegener’s granulomatosis, Infarction, Spleen, Case report

## Abstract

**Background:**

ANCA-associated vasculitis is a life-threatening, systemic autoimmune disease. There is an increased risk of organ infarction but in many cases this is asymptomatic. We described here the first reported case of PR3 vasculitis presenting with symptomatic bilateral renal wedge infarction.

**Case presentation:**

A 19-year old Caucasian woman with no past medical history presented on a number of occasions over a number of weeks with progressively more severe back pain, fevers and arthralgia. On the final presentation she was noted to have developed splinter haemorrhages and her blood tests revealed impaired renal function along with elevated inflammatory markers. She was subsequently found to have high titres of serum PR3 antibodies and focal necrotising glomerulonephritis on renal biopsy, consistent with a diagnosis of PR3 ANCA-associated vasculitis. Cross-sectional imaging revealed multiple wedge infarcts of her spleen and both kidneys, confirmed on contrast-enhanced ultrasound. Large vessel, cardiac and thrombophilic causes of thromboembolism were excluded. She was treated with high-dose corticosteroids and CD20 monoclonal antibodies (rituximab) and at time of writing, 4 months after initial presentation, has entered clinical remission.

**Conclusions:**

Here we describe the first reported case of PR3 vasculitis presenting with symptomatic renal wedge infarction. In patients with vasculitis who present with flank or back pain, infarction of abdominal organs should be considered in the differential.

Both splenic and renal infarctions are likely underdiagnosed in the setting of ANCA-associated vasculitis but may have clinical impact in contributing to infection risk and the degree or renal recovery, respectively.

## Background

Antineutrophil cytoplasmic antibody (ANCA)-associated vasculitis (AAV) is a rare autoimmune disease with increasing annual incidence, recently estimated at 20 per million per year. It is associated with antibodies to proteinase 3 (PR3) or myeloperoxidase (MPO) and is generally considered as a disease affecting small blood vessels (‘small vessel vasculitis’). PR3 antibodies are most commonly associated with granulomatous polyangiitis (GPA), with peak incidence in 50 to 70 year olds and affecting more men than women [1].

Symptoms and signs in GPA predominantly relate to granulomatous inflammation causing necrosis in capillaries, venules, arterioles and arteries. This leads to the renal and respiratory tract manifestations of rapidly progressive renal failure, pulmonary infiltrates or haemorrhage and upper respiratory dysfunction. Constitutional symptoms including weight loss and pyrexia are common and polyneuropathy may be a feature.

In this report, we detail an unusual case of GPA affecting a girl in her late teens, which presented with symptomatic splenic and renal infarction, with features of medium-vessel vasculitis.

## Case presentation

A 19 year old Caucasian woman presented to the emergency department (ED) with generalised myalgia, fevers and low back pain on the background of two weeks of coryzal symptoms. She had no significant past medical history and her only medication use was the combined oral contraceptive pill, taken for menorrhagia. She denied any history of illicit drug use nor any family history of coagulopathy, connective tissue or renal disease. She was a student at the local university and did not smoke or drink alcohol on a regular basis. She demonstrated no elicitable flank tenderness but did exhibit mild suprapubic tenderness. Her renal function was normal (Cr 68 μmol/L) and her inflammatory markers were elevated. Urine dip revealed moderate pyuria, 1+ proteinuria and heavy microscopic haematuria, which was attributed to menstruation (which had finished 24 h beforehand). She was mildly tachycardic (heart rate 110) and mildly hypertensive (blood pressure 138/88 mmHg) with a low-grade fever (37.5 °C). A presumptive diagnosis of urinary tract infection on the background of viral illness was made and after 24 h observation she was discharged on oral antibiotics. Her urine culture showed mixed growth, likely to represent perineal contamination.

She presented again to the ED one week later with ongoing symptoms. In addition she now complained of ocular pruritus and a mild cough, productive of white sputum. Her inflammatory markers remained elevated and her creatinine, whilst still in the normal range, was elevated compared to previous (Cr 86 μmol/L). Her urine dip was similar to previous. She was reassured and discharged with the diagnosis of viral infection.

**Fig. 1:**
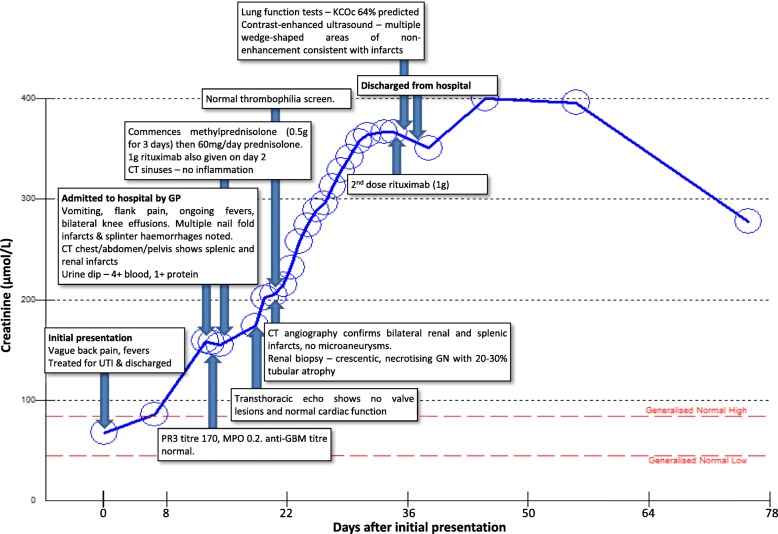
Clinical course and renal function (creatinine). UTI; urinary tract infection. CT; computed tomography. GP; general practitioner. PR3; proteinase 3. MPO; myeloperoxidase. Anti-GBM; anti-glomerular basement membrane. KCOc; corrected gas transfer (corrected for body mass index and contemporaneous blood haemoglobin)

One week later she was re-referred to hospital by her general practitioner due to ongoing lethargy, medium and small joint arthralgia, pyrexia, nausea and now bilateral flank pain. There was no history of haemoptysis. On admission she was tender in both flanks and in the suprapubic region. She now demonstrated splinter haemorrhages in the fingernail beds and tender proximal interphalangeal joints without overt synovitis bilaterally. She had bilateral knee effusions. Her admission creatinine was 159 μmol/L. The course of her renal function with major clinical events is summarised in Fig. 1. She had a microcytic anaemia (haemoglobin 94 g/L) with a mild thrombocytosis (platelet count 581 × 10^9^/L), leucocytosis (white cell count 27.3 × 10^9^/L) and elevated erythrocyte sedimentation rate (86 mm/hr). Urinalysis demonstrated moderate proteinuria (quantified as equivalent to 1.2 g/day) and heavy microscopic haematuria. The initial diagnosis was of possible sepsis arising from an abdominal or endocarditic source. She was commenced on broad-spectrum intravenous antibiotics and a computed tomography (CT) of her chest, abdomen and pelvis revealed “multiple wedge-like areas of low attenuation in both kidneys consistent with infarcts”. There were two areas of ground glass shadowing in the right upper lobe of the lung, suggestive of pulmonary haemorrhage, though subsequently pulmonary function testing was not consistent with this (Fig. 1).

An urgent transthoracic echo demonstrated normal cardiac size and function with no intracardiac thrombi or vegetations. Admission urine and serial blood culture were subsequently negative. Autoimmune serology yielded a cytoplasmic anti-neutrophil cytoplasmic antibody (cANCA) titre of > 1:40 with markedly elevated proteinase 3 (PR3) antibody and rheumatoid factor titres (170 U/mL [normal range < 3 U/mL] and 42 [normal range < 15 U/mL] respectively). Anti-nuclear antibodies, glomerular basement membrane antibodies, cryoglobulins, C3 and C4 levels were normal. Hepatitis B and C and Human Immunodeficiency Virus (HIV) serology was negative. On specific questioning it was revealed that both her mother and maternal aunt had a history of recurrent miscarriages: however a thrombophilia screen was negative (Table 1).

**Table 1:** Thrombophilia screen results

Test	Result	Comment
Thrombin time (pt)	14.8 s	Normal
Thrombin time (control)	16.6 s	Normal
Activated partial prothrombin time (A PTT)	27.8 s	Normal
Anti-thrombin III	110 IU/dL%	Normal
Protein C activity	164 IU/dL	High result
Protein S activity	> 150%	High result
Activated Protein C Resistance	None demonstrated	Normal
PTT Lupus anticoagulant screen	37.1 s	Normal
PTT LA ratio	1.03	Normal
DRVVT	45.35	Normal
DRVVT ratio	1.12 s	Normal
Actin FS APTT	27.00s	Normal
Lupus anticoagulant	Negative	Normal
Anti-cardiolipin	Negative	Normal
Anti-beta-2 glycoprotein-1 IgM	Negative	Normal

**Fig. 2:**
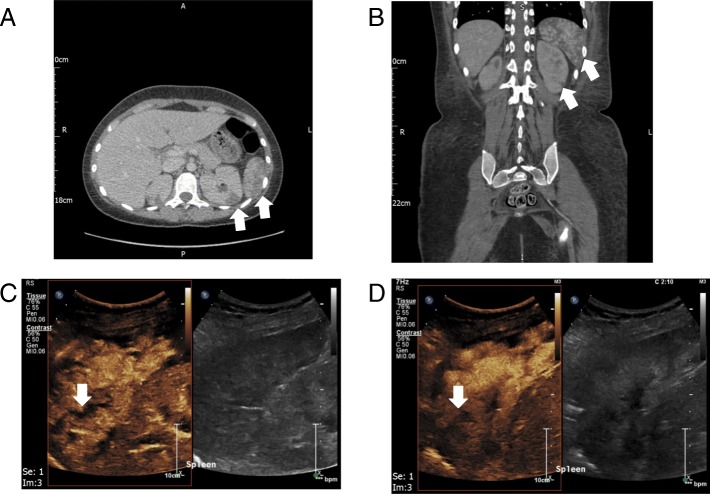
Imaging of splenic and renal infarcts. **a** Axial CT day 13 post-initial presentation, illustrating wedge-shaped renal and splenic infarcts (example indicated on all images with white arrow). **b** Coronal CT angiogram day 19 post-initial presentation. **c** and **d** Contrast-enhanced ultrasound (CEUS) demonstrating non-enhancing areas of spleen at 14 s (**c**) and 2 min 10 s (**d**) post-contrast injection

CT angiogram revealed normal large vessels but multiple bilateral renal infarcts and splenic infarcts (Fig. 2b), which, on retrospective review, were present on the admission CT (Fig. 2a). Due to the slightly atypical splenic distribution of the infarcts, sulphur hexafluoride microbubble (SonoVue, Bracco UK) contrast-enhanced ultrasound (CEUS) was performed, which confirmed the ischaemic nature of the splenic lesions, with > 50% normal splenic enhancement but multiple areas of wedge-shaped non-enhancement at 2 min post contrast (Fig. 2c and d). The patient was initially anticoagulated with high-dose low molecular weight heparin and was converted to oral anticoagulation with warfarin after renal biopsy.

Renal biopsy demonstrated a necrotising crescentic glomerulonephritis (crescents seen in 17 of 25 glomeruli), extensive mononuclear interstitial infiltrate, frequent tubular red cell casts and 30% interstitial fibrosis and tubular atrophy. There was no evidence of extraglomerular arteritis.

A diagnosis of PR3 AAV involving small and medium vessels was made and treatment was initiated (48 h after admission) with intravenous methylprednisolone followed by oral prednisolone. The patient subsequently received two doses of 1 g rituximab at a fortnightly interval. Renal function has continued to deteriorate throughout the investigative process and peaked at around 400 μmol/L. Once plateaued at this level the patient was discharged on a weaning course of corticosteroids.

On review in clinic around one month later the patient’s symptomatology was much improved and her creatinine was starting to fall (Fig. 1). Repeat PR3 titre was 22 U/mL. Most recent testing, 16 weeks after initial presentation, demonstrated a creatinine of 196 μmol/L, erythrocyte sedimentation rate of 27 mm/hr. and a mild leucocytosis (white cell count 16.1 × 10^9^/L). Systemic oral anticoagulation is intended to continue for six months after presentation.

## Discussion and conclusions

PR3 vasculitis is traditionally considered a disease of ‘small-to-medium vessels’, with a predilection for renal and pulmonary sites. In this report, we detail how the predominant presenting symptoms related to infarction of multiple abdominal organs, but without clinical or radiological evidence for a source of thromboembolism or a co-existent large- or middle-vessel pathology such as Behçet’s disease, aortitis or polyarteritis nodosa. In situ thrombi formation is also well-described in the setting of extrinsic vascular compression or blood vessel injury, thrombotic microangiopathy, low-flow states (such as cardiogenic shock), malignant hypertension, sickle cell anaemia and hypercoagulable states. All of these potential causes were excluded or felt to be sufficiently clinically unlikely as to not warrant further investigation.

The case’s use of the combined oral contraceptive pill is potentially relevant, as oestrogen-containing contraceptive use is associated with an increased risk of thrombosis [2]. However, it would be unlikely for a patient to develop multiple simultaneous oral contraceptive-associated in situ arterial infarcts in the absence of a genetic thrombophilic tendency, at the same time as presenting with a new diagnosis of AAV, suggesting that the vasculitis is a much more significant contributory factor.

Patients with active AAV demonstrate altered indices of coagulation and fibrinolysis, indicating a hypercoagulable state leading to a well-recognised increase in venous thromboembolic risk which does not clearly differ between MPO and PR3 vasculitis [3, 4]. Peripheral or abdominal arterial thrombosis in the setting of vasculitis is less well described, though there is a well-documented predisposition for cardiovascular events in active AAV [5]. Whilst clinical trials in this area have reported the incidence of venous thromboembolism, no major trials have reported specifically on the incidence of arterial thrombosis. The ‘Randomized Trial of Plasma Exchange or High-Dosage Methylprednisolone as Adjunctive Therapy for Severe Renal Vasculitis’ (MEPEX trial) reported a total of 5 ‘thrombosis’ adverse events but did not specify whether this represented arterial or venous disease [6]. The Wegener’s Granulomatosis Etanercept Trial (WGET) reported a single asymptomatic, radiologically detected, splenic infarction amongst 180 participants at enrolment [7]. This is likely an underestimate of prevalence and reflects the silent and clinically undetectable nature of infarcts in many cases [8–13]. In contrast, two historical post mortem case series of patients with granulomatous polyangiitis demonstrated splenic involvement in most or all cases, manifesting as splenomegaly, haemorrhage, capsular adhesion and infarction [14, 15]. Renal infarcts were described in two of six cases in one of these series [14].

The spleen is particularly predisposed to infarction due to occlusive infarction of distal parenchymal splenic arteries and arterioles, which are end-vessels that lack collaterals [11]. Clinically apparent splenic infarction appears to be a rarer phenomenon with few reported cases, but typically presenting with left sided abdominal pain [16, 17]. In contrast, in the case reported here the patient reported flank and back pain as the predominant presenting complaint, which probably represented symptomatic renal and splenic wedge infarction and was initially misdiagnosed as a urinary tract infection. We have identified only two previously reported cases of *ante mortem* presentation of renal cortical infarction in the context of multiorgan infarction in ANCA-associated vasculitis. Both were clinically silent and discovered incidentally, one in a young and one in an elderly patient, and the latter was associated with a poor outcome [11, 12]. The mechanism is presumed to relate to necrotising medium and small vessel inflammation with endothelial cell dysfunction and in situ thrombosis, with resultant downstream infarction [5].

The decision to anticoagulate the patient in this setting was a pragmatic one and discussed at length with clinical haematologists. There is no trial or clinical evidence to support this approach in the setting of AAV or autoimmune disease given the relative paucity of information regarding peripheral or abdominal vascular thrombosis in the absence of other factors such as cardiolipin antibodies or lupus anticoagulant.

Given the presumptive mechanism discussed above, the definitive treatment for the effectively pro-coagulant milieu should be to treat the underlying inflammation with appropriate immunosuppression. However, as this may take a number of days or even weeks to achieve maximal effect, it was felt appropriate to use systemic anticoagulation to reduce the risk of further, potentially catastrophic, vascular infarction in the interim. The decision to continue anticoagulation for a six month period was similarly a pragmatic one and reflected a) the potential duration of time to achieve remission of her AAV and b) extrapolation of guidelines relating to central thrombosis in other settings. Given the presence of endothelial cell dysfunction in AAV, the empirical use of antiplatelet agents may have been an acceptable, albeit similarly unevidenced, alternative.

In conclusion, splenic infarction is an underreported but important and frequent silent feature of AAV, typically complicating GPA. This is clinically relevant as many patients will receive heavy immunosuppression in order to control disease activity and may be further predisposed to infection by subsequent hyposplenism. Renal infarction in AAV appears to be rare but is equally important as infarction of renal cortex may have a potentially significant impact upon the subsequent level of renal function, beyond the damage caused by direct glomerular and tubular inflammation. The segmental nature of wedge infarction means that, unless extensive, the diagnosis is likely to be missed by renal biopsy.

We describe the first reported case of symptomatic bilateral renal cortical infarction in association with partial splenic infarction, with a good outcome following treatment with corticosteroids, rituximab and anticoagulant therapy. There should be a low threshold for abdominal imaging in patients with AAV presenting with back or flank pain as the presence of either renal or splenic infarction may have clinical consequences.

## Abbreviations

AAV: ANCA-associated vasculitis
ANCA: Anti-neutrophil cytoplasmic antibody
cANCA: Cytoplasmic anti-neutrophil cytoplasmic antibody
CEUS: Contrast-enhanced ultrasound
CT: Computed tomography
ED: Emergency Department
GPA: Granulomatous polyangiitis
HIV: Human Immunodeficiency Virus
MPO: Myeloperoxidase
PR3: Proteinase 3
